# Plasticity of adipose tissue in response to fasting and refeeding in male mice

**DOI:** 10.1186/s12986-016-0159-x

**Published:** 2017-01-05

**Authors:** Hao-Neng Tang, Chen-Yi Tang, Xiao-Fei Man, Shu-Wen Tan, Yue Guo, Jun Tang, Ci-La Zhou, Hou-De Zhou

**Affiliations:** 1 Department of Endocrinology and Metabolism, National Clinical Research Center for Metabolic Diseases, The Second Xiangya Hospital, Central South University, 139 Ren-Min Middle Road, Changsha, Hunan 410011 China; 2 Department of Laboratory Medicine, The Second XiangYa Hospital, Central South University, Changsha, Hunan 410011 China

**Keywords:** Obesity, Fasting and refeeding, Plasticity, Adipose tissue, Fat distribution

## Abstract

**Background:**

Fasting is the most widely prescribed and self-imposed strategy for treating excessive weight gain and obesity, and has been shown to exert a number of beneficial effects. The aim of the present study was to determine the exact role of fasting and subsequent refeeding on fat distribution in mice.

**Methods:**

C57/BL6 mice fasted for 24 to 72 h and were then subjected to refeeding for 72 h. At 24, 48 and 72 h of fasting, and 12, 24, 48 and 72 h of refeeding, the mice were sacrificed, and serum and various adipose tissues were collected. Serum biochemical parameters, adipose tissue masses and histomorphological analysis of different depots were detected. MRNA was isolated from various adipose tissues, and the expressions of thermogenesis, visceral signature and lipid metabolism-related genes were examined. The phenotypes of adipose tissues between juvenile and adult mice subjected to fasting and refeeding were also compared.

**Results:**

Fasting preferentially consumed mesenteric fat mass and decreased the cell size of mesenteric depots; however, refeeding recovered the mass and morphology of inguinal adipose tissues preferentially compared with visceral depots. Thermogenesis-related gene expression in the inguinal WAT and interscapular BAT were suppressed. Mitochondrial biogenesis was affected by fasting in a depot-specific manner. Furthermore, a short period of fasting led to an increase in visceral signature genes (*Wt1, Tcf21*) in subcutaneous adipose tissue, while the expression of these genes decreased sharply as the fasting time increased. Additionally, lipogenesis-related markers were enhanced to a greater extent greater in subcutaneous depots compared with those in visceral adipose tissues by refeeding. Although similar phenotypic changes in adipose tissue were observed between juvenile mice and adult mice subjected to fasting and refeeding, the alterations appeared earlier and more sensitively in juvenile mice.

**Conclusions:**

Fasting preferentially consumes lipids in visceral adipose tissues, whereas refeeding recovers lipids predominantly in subcutaneous adipose tissues, which indicated the significance of plasticity of adipose organs for fat distribution when subject to food deprivation or refeeding.

**Electronic supplementary material:**

The online version of this article (doi:10.1186/s12986-016-0159-x) contains supplementary material, which is available to authorized users.

## Background

Obesity, which is associated with a cluster of metabolic abnormalities, has become an international public health issue that affects the quality of life, increases the risk of illness and shortens life span. Obesity has a multifactorial nature resulting from genetic, epigenetic, behavioural, physiological, environmental and sociocultural factors that lead to an imbalance between energy intake and expenditure over an extended time period [1–4]. Strategies to manage obesity or overweight include dieting, physical activity, pharmacotherapy and surgery [2]. The current obesity epidemic has focused a great deal of attention on mechanisms that control the energy balance [1, 5]. Eating as a main form of energy intake is closely related to the development of obesity. Therefore, as an effective way to restrict energy intake, fasting or dieting can play an important role in preventing the development of obesity and related metabolic disorders [6–9]. Fasting and dieting induce specific molecular and metabolic adaptations in most organisms, and are the most widely prescribed and self-imposed strategies to treat excessive weight gain and obesity. Fasting and dieting exert a number of beneficial effects, including the prolongation of life span, in rodents and humans [9–12]. There are different forms of fasting, including intermittent fasting and periodic fasting lasting several days or longer (two or more weeks) [9]. Rather than reducing daily total caloric intake, intermittent fasting (IMF) has received attention as a possible approach for long-term weight loss [10, 13]. However, there are some controversies regarding the effects of fasting and refeeding on weight regain [14, 15]. Many individuals that lose weight are unable to maintain the weight loss over time [6]. In addition, weight regain after fasting and subsequent ad libitum refeeding result in accelerated fat storage in adipose tissue. The “catch-up growth” might even increase the risk of developing obesity [16, 17]. Whether fasting is a causative factor in subsequent weight gain and contributes to the current obesity epidemic has been a subject of considerable debate [8]. Consequently, how to fast reasonably and efficiently to control weight and affect fat distribution of adipose tissues are of great significance for obesity prevention and intervention.

Adipose tissues are divided into two types: white adipose tissue (WAT) and brown adipose tissue (BAT). WAT generally develops in discrete anatomical depots, identified as subcutaneous adipose tissue (SAT) and visceral adipose tissue (VAT); the distribution of fat is closely linked to metabolic disease risk [18, 19]. Emerging evidence has confirmed that the accumulation of VAT is closely related to the metabolic complications of obesity and cardiovascular disease [20–23]. By contrast, the expansion of subcutaneous adiposity shows little or even an inverse correlation with disease risk [23–25]. Fat distribution in various body locations affects the development and progression of metabolic diseases more than does total fat mass [26]. Pharmacological therapy for obesity has also been suggested to specifically target VAT loss [20], because the benefit of reducing VAT is significant for obesity, especially obesity associated with metabolic abnormalities [27]. The results of many clinical trials have verified that almost all forms of weight loss affect visceral fat more than subcutaneous fat [20, 28–30]. Several studies have reported that mobilization of the visceral depot appears to be faster than that of subcutaneous fat during food deprivation [31]; however, the molecular mechanisms are not fully understood. In addition, in view of the existence of the contradiction between intermittent fasting and “catch-up growth” of fat, which type of adipose tissue is restored preferentially during weight regain after fasting, and whether refeeding influences fat distribution should be explored.

In addition, age-related changes occur in adipose tissue [32] and research continues to validate that various fat depots are affected differently by aging [33, 34], which results in the redistribution of adipose tissue from subcutaneous to visceral locations [35, 36]. Furthermore, metabolic syndrome often tends to occur in adults rather than in adolescents, while metabolic benign obesity is the main type of obesity occurring in adolescents [37–39]. Therefore, age-related changes in body fat distribution might be essential factors for the development of obesity [40].

To determine the exact role of fasting and subsequent refeeding on fat distribution in mice, herein, we compared the morphological, histological and gene expression alterations in various adipose tissues after long-time fasting and subsequent refeeding in mice. We also compared the phenotypical differences in adipose tissues between juvenile mice and adult mice subjected to fasting and refeeding.

## Methods

### Animals

All animal experimental procedures were approved by the Animal Care Committee of the Central South University (Changsha, China). Experiments were carried out on juvenile (1-month-old) and adult (3-months-old) male C57BL/6 J mice obtained from the Model Animal Research Center of the Central South University. Animals were housed individually in ventilated Plexiglas cages within a pathogen-free barrier facility and maintained under a 12-h light/12-h dark cycle with a standard rodent chow (7.0% fat, 18.7% protein, 64.7% carbohydrate, and 5.0% fiber, both diets were supplement with a similar mix of minerals and vitamins; According to the standards of American Institute of Nutrition, AIN-93G). For fasting, the mice were housed individually at room temperature (22 °C ~ 25 °C) with water only. 1-month-old (*n* =54) and 3-months-old (*n* = 48) mice were assigned randomly to the following groups: control animals fed ad libitum (Con, *n* = 6–12), animals fasted for 12 h (F12, *n* = 5), 24 h (F24, *n* = 6), 48 h (F48, *n* = 6) and 72 h (F72, *n* = 6), respectively, or fasted for 72 h and subsequently refed for 12 h (R12, *n* = 6), 24 h (R24, *n* = 6), 48 h (R48, *n* = 6) and 72 h (R72, *n* = 6), respectively. Food intake and body weight were measured daily early in the light cycle before the mice were sacrificed. All animals were subjected in all protocols at room temperature (22 °C ~ 25 °C).

### Serum and tissue collection

After treatment, mice were anesthetized with isoflurane and blood was taken from the retro-orbital sinus. Blood samples were allowed to clot and were subsequently centrifuged (3500 rpm, 5 min, 4 °C). Serum was frozen at −20 °C until analysis. Adipose tissues in present study were extracted at 9 am every day. Various adipose depots, including major subcutaneous WATs, -inguinal WAT (ingWAT), two representative visceral WATs, -mesenteric (mWAT) and -epididymal (eWAT) WAT; and -interscapular BAT (iBAT) were sampled, weighed, immediately frozen in liquid nitrogen and stored at −80 °C for later RNA isolation and analysis. The remaining fat tissues from each location were carefully excised and weighed.

### Biochemical assays

Concentrations of glucose (GLU), β-hydroxybutyrate (BK), triglyceride (TG), total cholesterol (TC), high density lipoprotein cholesterol (HDL-C), low density lipoprotein cholesterol (LDL-C), non-esterified fatty acid free fatty acid (NEFA),total protein (TP) albumin (AlB) in serum were measured in the Department of Laboratory Medicine, The Second Xiangya Hospital, using routine diagnostic tests.

### Hematoxylin and eosin (H&E) staining and adipocyte size measurements

Adipose tissues rinsed with saline solution were fixed in 10% neutral formalin buffered solution, embedded in paraffin, cut into 10 mm sections and stained with H&E for morphological observation. Adipocyte cell sizes in the adipose tissue were measured as described previously [41]. Briefly, images were captured using a light microscope (Olympus, Center Valley, PA, USA) equipped with a digital camera. Three to five fields per slide were chosen randomly by one blinded evaluator for imaging. Images were analyzed using Image-Pro Plus software. Cell areas (μm^2^) were measured and averaged for each section.

### Transmission electron microscopy (TEM)

Transmission electron microscopy investigation was performed using an H-7600 (Hitachi, Japan) at the Electron Microscopy Center of the Central South University. Tissue samples were minced to less than 1 mm in each dimension, followed by TEM sample preparation and imaging [42]. Briefly, fat tissues were excised into small pieces (<1 mm^3^) and fixed with 2.5% glutaraldehyde (0.1 M phosphate buffer, pH 7.4) for 4 h, before being post-fixed in 1% osmium tetroxide. Specimens were then dehydrated with graded acetone solutions, and embedded in an Epon-Araldite mixture. Thin sections were stained with uranyl acetate and lead citrate. The instrument was operated at 60–80 KV. Photomicrographs were taken at 15,000× magnification. The number of mitochondria was analyzed from in five to eight randomly delineated micrographs per group using Image-Pro Plus software.

### Quantitative real-time PCR (qPCR)

Total RNA was extracted from frozen adipose tissue using the Trizol Reagent, according to the manufacturer’s instructions (Thermo Fisher Scientific). Reverse transcription was performed to synthesize cDNA using an RT Kit (Takara, Otsu, Japan). The primers used for real-time qPCR are shown in Table 1. QPCR was performed on a Light Cycler 480 Real time-PCR Detection System (Roche, Basel, Switzerland) using SYBR Green PCR master mix (Takara), following the manufacturer’s instructions. The relative mRNA expression was normalized to that of β-actin and expressed as 2^−ΔΔCT^ relative to the control group. Additionally, a positive control of ki67 was stet up to assure negativity in the experiments.

**Table 1:** Real-Time RT-PCR Primer Sequences

Gene	Forward primer (5’ to 3’)	Reverse primer (5’ to 3’)
β-actin	TCG TTAC CAC AGG CAT TGT GAT	TGC TCG AAG TCT AGA GCA AC
*Ucp-1*	AAGCGTACCAAGCTGTGCGA	AGAAAAGAAGCCACAAACCCTT
*Pgc-1α*	TGAACGCACCTTAAGTGTGGAA	GGGTTATCTTGGTTGGCTTTATGA
*Atgl*	AACACCAGCATCCAGTTCAA	GGTTCAGTAGGCCATTCCTC
*Hsl*	TGAGATGGTAACTGTGAGCC	ACTGAGATTGAGGTGCTGTC
*Pparα*	ATCCACGAAGCCTACC	CACACCGTACTTTAGCAAG
*Cpt-1 m*	TGCCTTTACATCGTCTCCAA	GGCTCCAGGGTTCAGAAAGT
*Wt1*	AGCTGTCCCACTTACAGATGC	CCTTGAAGTCACACTGGTATGG
*Tcf21*	CATTCACCCAGTCAACCTGA	TTCCTTCAGGTCATTCTCTGG
*Ki67*	GAATGAATGCAGAAATCAGCGGTA	GATCATGGATGACGCTGTGAGAA
*Pcna*	CGTGAACCTCACCAGCAT	ATTTGGAGCTTCAAACACT
*Pparγ*	ATGGTTGACACAGAGATGC	GAATGCGAGTGGTCTTCC
*C/ebpα*	CAAGAACAGCAACGAGTACCG	GTCACTGGTCAACTCCAGCAC
*Srebp-1c*	GGAGCCATGGATTGCACATT	AGGAAGGTTCCAGAGAGGA
*Fas*	CAC ACA CAA TGG ACC CCC AG	CAG AGG TGT TCG GCT TCA GG

### Statistical analyses

Statistical analysis was performed by using SPSS software, version 17.0. All values are presented as the mean ± SEM, unless otherwise indicated. Statistical analyses consisted of two-tailed unpaired Student’s *t* test and one-way ANOVA with post hoc Tukey’s test, with *p* <0.05 being considered statistically significant.

## Results

### Effects of fasting and refeeding on body weight, adipose tissue mass and morphology

**Fig. 1:**
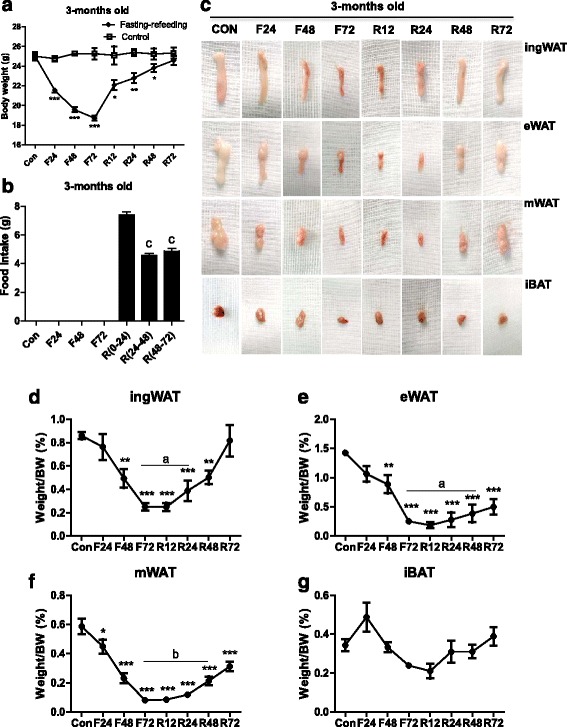
Alteration of body weight and fat mass in mature mice subjected to fasting and refeeding. 3-months-old mice were fasted for 24, 48 and 72 h (F24, F48 and F72) respectively, and then fed again for 12, 24, 48 and 72 h (R12, R24, R48 and R72), respectively, after 72 h of fasting. Body weights (**a**), food intakes (**b**) and tissue appearance (**c**) of inguinal white adipose tissue (ingWAT), epididymal WAT ((eWAT), mesenteric WAT (mWAT) and interscapular BAT (iBAT) of mice during different fasting and feeding conditions were detected. Weights of adipose tissues (ingWAT, eWAT, mWAT, iBAT) expressed as a percentage of body weight in adult mice (**d**-**g**) were also analyzed. All data are presented as the mean ± SEM. * p < 0.05; ** p < 0.01; *** p < 0.001 compared with control mice (Con); ^*a*^ *p <0.05,* ^*b*^ *p <0.001* compared with 72 h-fasting; ^*c*^ *p <0.01* compared with 24 h-refeeding (one-way ANOVA)

We first studied the effect of fasting and refeeding on body weight and fat mass of adult male mice (3-months-old). Fasting markedly reduced their body weights and the lost body mass was restored after 3 days of refeeding (Fig. 1a). It is worth noting that the speed of weight recovery was higher during the initial phase of refeeding (24 h) than during the latter stages (Fig. 1a). Food intake during 24 h of refeeding was much greater than at 48 and 72 h of refeeding (Fig. 1b), which suggested that the rapid increase of body weight might be attributed to hyperphagia at the initial phase of refeeding.

Representative images of various kinds of adipose tissue are shown in Fig. 1c. The percentages of body weight of the different adipose tissue depots were also determined (Fig. 1d-g). After fasting for 24 h, the percentage of visceral WATs was reduced (eWAT, −33.5%, *p* >0.05; mWAT, −37.6%, *p* <0.05) (Fig. 1c, e, f), while there were no changes in the masses of the ingWAT and iBAT (Fig. 1c, d, g). With increasing fasting time, both the percentage of ingWAT and visceral WATs were reduced, with the fat mass being almost totally consumed (ingWAT, −71%, *p* <0.0001; eWAT, −82.5%, *p* <0.0001; mWAT, −87.2%, *p* <0.0001) after fasting for 72 h (Fig. 1c, d, e, f). We also found that iBAT only decreased by 30% (*p* >0.05) (Fig. 1g). After refeeding, the restoration speed of fat masses showed differences between various WAT depots. The recovery of visceral WATs was much slower than that of the ingWAT and the latter increased by 54.8% (*p* <0.05) compared to the level after 72-h of fasting (Fig. 1c, d). After refeeding for 72 h, the percentage of ingWAT recovered to normal levels (104.7% of control), while visceral WATs were only restored to half the control levels (eWAT, −47.6% of control, *p* <0.0001; mWAT, −57.6% of control, *p* <0.0001) (Fig. 1d, e, f). For the iBAT, there was no significant difference between the control and 72 h of refeeding, despite a tendency to decrease (*P* >0.05) (Fig. 1c, g).

**Fig. 2:**
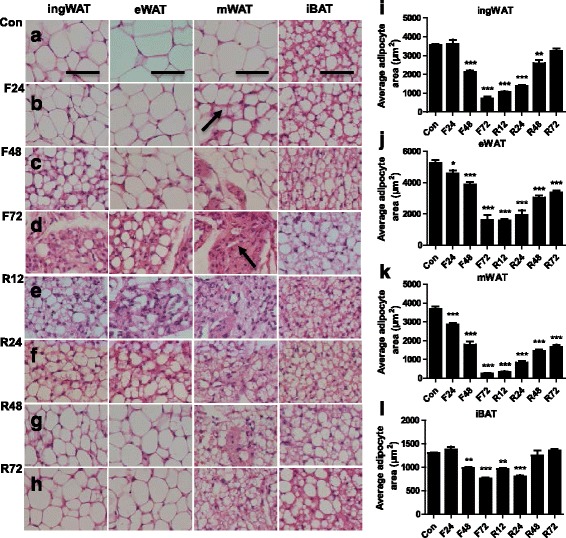
Histomorphological alterations of adipose tissue in adult mice subjected to fasting and refeeding. The effects of fasting and refeeding on histomorphological alterations of various adipose tissues in adult mice (**a**-**h**; arrow in panel **b** points to the ‘multilocular’ fat cells; arrow in panel **d** points to the slimmed adipocytes). The average area of adipocytes (μm^2^) in every 100-mm^2^ area range of various adipose tissues was quantified using Image Pro Plus software (**i**-**l**) (*n* = 6-8). Scale bar represents 100 μm, all data are presented as the mean ± SEM. * p < 0.05; ** p < 0.01; *** p < 0.001 compared with control (one-way ANOVA)

We next tested the influences of fasting and refeeding on the histomorphologies of various adipose tissues (Fig. 2). Fasting for 24 h caused a moderate decrease in epididymal cell size, while the average adipocyte area in the mesenteric depots was markedly reduced (Fig. 2b, j, k). A proportion of ‘multilocular’ fat cells mixed with regions of ‘unilocular’ fat cells were readily observed in the mWAT (Fig. 2b). However, no apparent changes in the ingWAT and iBAT morphologies were observed until fasting had lasted for 48 h (Fig. 2b, i, l). Completely delipidized adipocytes [43] were observed near apparently unaffected unilocular cells in the mWAT as fasting continued (Fig. 2c, d). The typical morphologies of WAT were almost completely suppressed and a variable amount of slimmed cells [43] were present in the WAT, especially in the mWAT when fasting for 72 h (Fig. 2d). After refeeding for 12 h, the average adipocyte area of the ingWAT increased quickly, while no apparent changes were observed in the visceral WAT (Fig. 2e, i, j, k). As the refeeding time increased, the adipocytes area expanded and the proportion of ‘multilocular’ fat cells decreased in the ingWAT (Fig. 2f-i,). Compared with the ingWAT, the recovery of the morphologies of visceral depots was much later and slower (Fig. 2e-h, j, k). After refeeding for 72 h, the adipocytes area of the ingWAT was similar to normal levels (Fig. 2h, i), whereas the area in the visceral depots was much smaller than that before fasting (Fig. 2h, j, k). Moreover, some atypical white adipocytes were observed in the visceral depots compared with adipocytes in the subcutaneous depots (Fig. 2h). These results revealed that the mesenteric depot had a greater tendency to decrease after fasting, followed by the epididymal depot and finally by the inguinal depot, whereas these fat depots were restored in the reverse order after refeeding.

### Effects of fasting and refeeding on serum biochemical parameters

After fasting, the serum concentrations of GLU and TG in adult male mice decreased significantly with extended fasting time (Table 2). There was a significant increase in both the serum GLU and TG levels after refeeding for 12 h; their levels returned to normal after refeeding for 72 h. However, β-hydroxybutyrate and NEFA levels in the serum increased significantly after fasting and then recovered to the normal levels after refeeding. Fasting and refeeding had little impact on TC and HDL-C levels, while the LDL-C level changed markedly after refeeding. These data revealed that there were no distinct plasma glucose abnormalities and dyslipidemias when adult mice were subjected to 72 h of fasting and subsequent 72 h of refeeding. In order to assess the nutritional status, ALB and TP concentrations were also determined. There were no significant changes until fasted up to 72 h (Table 2).

**Table 2:** Serum concentrations of metabolites in mature mice (3-months-old) subjected to fasting and refeeding

	CON	F24	F48	F72	R12	R24	R48	R72
GLU	10.28 ± 0.91	4.5 ± 0.54***	3.96 ± 1.18***	5.97 ± 2.67***	10.67 ± 1.18	8.29 ± 0.68*	9.2 ± 0.95	9.59 ± 1.62
BK	0.16 ± 0.07	1.38 ± 0.4***	2.51 ± 0.3***	1.22 ± 1.01***	0.18 ± 0.11	0.1 ± 0.02	009 ± 0.02	0.11 ± 0.04
TG	1.36 ± 0.34	1.5 ± 0.4	1.57 ± 0.19	0.83 ± 0.19	2.66 ± 1.0**	2.17 ± 1.17*	1.88 ± 0.72	1.32 ± 0.52
CHOL	2.4 ± 0.86	2.57 ± 0.21	2.87 ± 0.51	3.44 ± 0.74*	3.47 ± 1.02**	3.16 ± 0.66	2.99 ± 0.52	2.42 ± 0.74
NEFA	1.39 ± 0.53	1.76 ± 0.49	2.9 ± 0.36***	1.42 ± 0.0.36	2.75 ± 0.17***	1.33 ± 0.41	1.49 ± 0.13	1.15 ± 0.42
HDL-C	1.82 ± 0.19	1.99 ± 0.18	2.43 ± 0.13	2.59 ± 0.52**	2.72 ± 0.15**	2.24 ± 0.4	2.12 ± 0.08	1.63 ± 0.55
LDL-C	0.2 ± 0.07	0.16 ± 0.02	0.19 ± 0.06	0.32 ± 0.1*	0.64 ± 0.059***	0.47 ± 0.11***	0.65 ± 0.11***	0.3 ± 0.12
ALB	17.49 ± 1.17	17.07 ± 0.81	16.97 ± 0.29	16.13 ± 0.21*	16.4 ± 0.53	15.6 ± 0.44	17.03 ± 0.15	17.04 ± 1.46
TP	50.96 ± 3.03	51.07 ± 1.71	50.1 ± 1.06	46.53 ± 0.9*	46.2 ± 1.14*	46.3 ± 1.27*	52.43 ± 0.59	50.8 ± 4.21

All data are presented as the mean ± SEM. Statistically significant differences: * p <0.05; ** p <0.01; *** p <0.001; (One-way ANOVA) CON = control adult mice, fed ad libitum; F24, F48 and F72 = adult mice fasted for 24, 48 and 72 h, respectively; R12, R24, R48 and R72 = adult mice fasted for 72 h and subsequent fed again for 12, 24, 48 and 72 h, respectively (*n* = 6–8 animals in each group)

### Fasting affects mitochondrial biogenesis and inhibits thermogenesis-related gene expression in a depot-specific manner

**Fig. 3:**
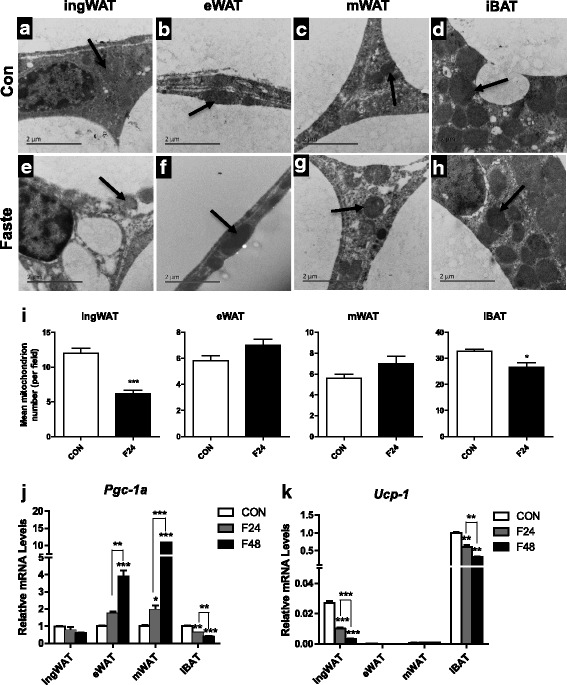
Effects of fasting on mitochondrial biogenesis and expression of thermogenesis-related genes in various adipose tissues. Representative transmission electron microscopy (TEM) images of various adipose tissues of adult mice fed ad libitum (**a**-**d**) or 24 h-fasting (**e**-**h**) mice (arrows indicate mitochondria). Scale bar, 2 μm. Mitochondrial numbers were decreased in ingWAT and iBAT, but increased in eWAT and mWAT after 24 h of fasting compared with the control (**i**). Differential expressions of mitochondrial biogenesis related gene (*Pgc-1α)* and thermogenic gene (*Ucp-1)* in various depots from ad libitum fed or fasted mice (**j**) were observed (*n* = 6). All data are presented as the mean ± SEM. * p < 0.05; ** p < 0.01; *** p < 0.001

To further understand the ‘multilocular’ fat cells that were observed at the initial stage of fasting, TEM was used to assess the ultrastructure of adipocytes in various fat depots (Fig. 3). The results showed that 24 h of fasting resulted in a significant decrease in the number of mitochondria in the ingWAT and iBAT (ingWAT, *p* <0.0001; iBAT, *p* <0.05, respectively, Fig. 3a, d, e, h, i). The number of mitochondria in visceral fat cells tended to increase after 24 h of fasting, although such changes did not show statistical significance (*p* >0.05, Fig. 3b, c, f, g, i). In accordance with the changes in mitochondrial number, the mRNA levels of *Pgc1α* (Fig. 3j) were inhibited in the iBAT (*p* <0.01) and showed a tendency to decrease in the ingWAT (*p* >0.05). By contrast, the expression of *Pgc1α* was elevated in the eWAT (*p* <0.05) and mWAT (Fig. 3j) (*p* <0.05). These results suggested that food deprivation affects mitochondrial biogenesis in a depots-specific manner. We further studied the expressions of thermogenesis-related genes in various fat depots under fasting conditions. The results showed that the mRNA levels of *Ucp-1* in the iBAT and ingWAT depots decreased sharply under fasting conditions, in a time-dependent manner (Fig. 3k). However, the expression of *Ucp-1* was almost undetectable in the eWAT and mWAT depots when the mice were fed a normal diet or subjected to fasting (Fig. 3k), suggesting that fasting restrains thermogenesis mainly in the iBAT and ingWAT.

### Fasting drives a reversible visceral-like phenotype of subcutaneous adipose tissue

**Fig. 4:**
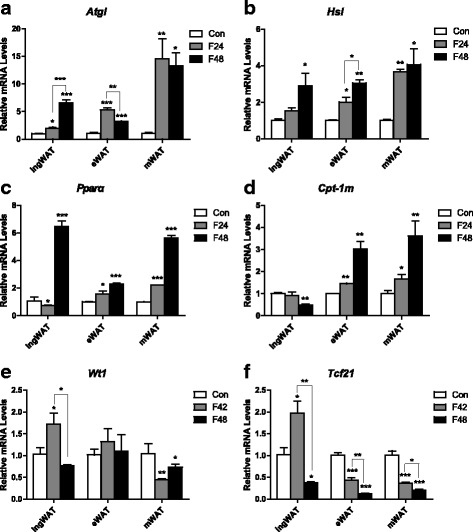
Expression of lipid metabolism-related genes and visceral signature genes of various white fat depots in adult mice under different feeding conditions. Differential expressions of lipid mobilization related genes (*Atgl, Hsl*) (**a**, **b**), fatty acid oxidation related genes (*Pparα, Cpt-1 m*) (**c**, **d**), and visceral signature genes (*Wt1, Tcf21*) (**e**, **f**) in inguinal, epididymal and mesenteric adipose tissues from ad libitum fed or fasted mice (**j**) were observed (*n* = 6–8). (*n* = 6–8). All data are presented as the mean ± SEM. * p < 0.05; ** p < 0.01; *** p < 0.001; (one-way ANOVA)

To further understand the exact effects of fasting on lipid metabolism in various fat depots, the expressions of genes involved in lipid mobilization and fatty acid oxidation were investigated. The expressions of the *Agtl* and *Hsl* genes were affected at the transcriptional level by starvation, although differences between depots were also observed (Fig. 4a, b). Fasting for 24 h led to an approximately 15-fold increase in *Agtl* mRNA levels and a 4-fold increase in *Hsl* mRNA levels in the mWAT (Fig. 4a, b). The expressions of the same genes were also elevated in the ingWAT, but to a lesser extent (Fig. 4a, b). When subject to fasting for 48 h, the mRNA levels of *Agtl* and *Hsl* continued to increase in the ingWAT; however, the expression of *Hsl* showed a much smaller increase (eWAT, *p* <0.05; mWAT, *p* >0.05) and the expression of *Agtl* even showed a tendency to decrease in visceral depots (eWAT, *p* <0.01; mWAT, *p* >0.05) compared with fasting for 24 h. The expressions of fatty acid oxidation-related genes were also affected in a depot-specific manner by fasting (Fig. 4c, d). *Pparα* mRNA levels were higher after 24 h of fasting in visceral depots (eWAT, *p* >0.05, mWAT, *p* <0.05 Fig. 4c) and continued to increase when fasted for 48 h (eWAT, *p* <0.01, mWAT, *p* <0.05, vs. 24 h of fasting, Fig. 4c). In contrast, the expression of *Pparα* showed a tendency to decrease in the ingWAT (*p* >0.05, Fig. 4c) and then markedly increased after 48 h of fasting (*p* <0.01, Fig. 4c). Notably, the mRNA levels of *Cpt-1 m* showed the same tendency as *Pparα*, except that the expression remained lower after 48 h of fasting in the ingWAT (*p* <0.01, Fig. 4d).

Recent research reported that fasting drives visceral-like phenotype switches in the ingWAT [23]. However, the fasting time in that study was limited to 24 h. Thus, we detected the expression of several visceral signature genes (e g. Wilms tumour 1 (*Wt1*), transcription factor 21(*Tcf21*)). Our data showed that although much lower than in the visceral adipose tissue, the mRNA levels of *Wt1* and *Tcf21* in the ingWAT were significantly upregulated in after fasting for 24 h (*p* <0.05, Fig. 4e, f). Surprisingly, the mRNA levels of these visceral signature genes were dramatically reduced as the fasting time extended to 48 h (*p* <0.05, *p* <0.001 vs. 24-h fasting, respectively. Fig. 4e, f). These results imply that the visceral depot of subcutaneous adipose tissue might contribute to the mobilization of white adipose tissue.

### Refeeding enhances adipogenesis in a depot-specific manner

**Fig. 5:**
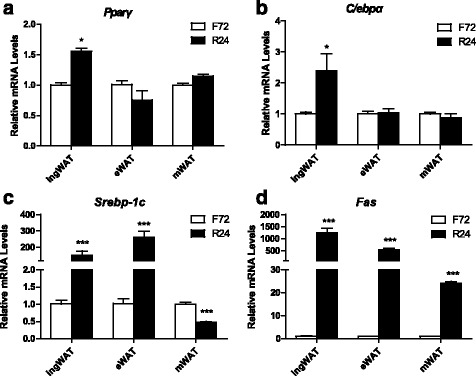
Effects of refeeding on lipogenesis of various white adipose tissues in adult mice. Differential expressions of lipogenesis-related transcription factors (*Pparγ,C/ebpα, Srebp-1c*) (**a**-**c**) and de novo fatty acid synthesis -related gene (*Fas*) (**d**) in inguinal, epididymal and mesenteric adipose tissue from adult mice subjected to 72 h-fasting and 24 h-refeeding (n =5–8). All data are presented as the mean ± SEM. * p < 0.05; ** p < 0.01; *** p < 0.001; (one-way ANOVA)

Given that the body weight and the inguinal fat content were restored quickly at the initial stage of refeeding, we investigated adipogenesis at this stage. Thus, mRNA abundances of proliferation-related genes (*Ki67, Pcna*), transcription factors involved in differentiation (*Pparγ, C/ebpα, Srebp-1c*) and a lipogenesis-related marker (*Fas*) were measured. The expressions of *Ki67* gene and *Pcna* gene were practically undetectable under fasting or under refeeding conditions. Significant increases in the mRNA expression levels of *Pparγ* and *C/ebpα* in the ingWAT were observed after 24 h of refeeding compared with those after 72 h of fasting (*p* <0.05). By contrast, no significant changes in these markers were observed in visceral depots (Fig. 5a, b). The results also revealed that the ingWAT displayed a remarkably increase in *Srebp-1c* mRNA under refeeding conditions (approximately 240-fold vs. 72 h of fasting, *p* <0.0001, Fig. 5c). In contrast, the same gene decreased in the mWAT (*p* <0.0001, Fig. 5c). The expression of the *Fas* gene increased by more than 1200-fold in the ingWAT, while its expression was elevated to a lesser extent in the visceral adipose tissue (Fig. 5d). These data suggested that an obvious difference in adipogenesis between the subcutaneous and visceral adipose tissues under refeeding conditions.

### Similar phenotypic changes in the adipose tissue between juvenile mice and adult mice subjected to fasting and refeeding

**Fig. 6:**
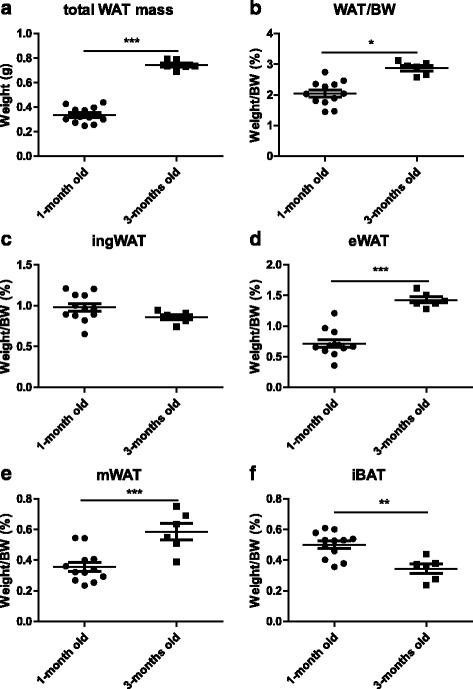
Differences in fat mass and fat distribution between adult and juvenile mice. Adult mice (3-months-old) and juvenile mice (1-month-old) were both fed ad libitum. Total white adipose tissue (ingWAT + eWAT + mWAT) mass (**a**), percentage of total white adipose tissues mass in body weight (**b**), and percentage of various fat depots in body weight (**c**-**f**) in both types of mice were detected (n = 6–12) . All data are presented as the mean ± SEM. * p <0.05; ** p <0.01; *** p <0.001; (Student’s *t*-test)

Given the interesting phenotypic changes in adult mice after fasting and refeeding, we further investigated whether these changes occurred in juvenile mice. We first compared the differences in fat distribution between juvenile mice (1-month-old) and adult mice (3-months-old). As expected, the data showed that both the fat mass and the percentage of body fat of the adult mice were higher than those in the juvenile mice (Fig. 6a, b). Age-related alterations in percentage of body fat in various fat depots were evident (Fig. 6c-f). The percentage of iBAT in the 3-month-old mice was lower than that in the 1-month-old mice (Fig. 6f), while the percentage of visceral WAT (including eWAT and mWAT) was significantly higher in the aged compared with the younger mice (Fig. 6d, e). Surprisingly, there was no remarkable difference in the percentage of ingWAT between these two groups of mice (Fig. 6c).

We then performed the fasting-refeeding experiment on the 1-month-old mice. Serum biochemical parameters showed no noteworthy differences between the juvenile and adult mice (Additional file 1: Table S1), except that the serum GLU levels tended to increase and TG levels tended to decrease in juveniles after refeeding for 72 h (Additional file 1: Table S1). The effects of fasting and refeeding on body weight, fat mass and percentage of body fat, as well as the morphology of the 1-month-old mice, were similar to those of the 3-month-old mice (Additional file 2: Figure S1 A-C; Additional file 3: Figure S2). However, alterations of fat depots appeared earlier and were more sensitive in the juvenile mice when subjected to fasting or refeeding (Additional file 2: Figure S1 C-F, Additional file 3: Figure S2). Mobilization of mesenteric WAT even occurred after 12 h of fasting (Additional file 2: Figure S1 C, S1 F, Additional file 3: Figure S2 B). After refeeding, 12 h of refeeding led to a significant increase in fat mass and an enlarged fat cell size in the ingWAT, which suggested subcutaneous adipose tissue tended to recover more quickly (Additional file 2: Figure S1 C, S1 D; Additional file 3: Figure S2 F, S2 J). After refeeding for 72 h, the recovery of fat mass in the 1-month-old mice was greater than that in the 3-month-old mice, although neither the eWAT nor mWAT of the juvenile mice were restored to their normal levels (eWAT, −88.9% vs. control, *p* >0.05; mWAT, −73.6% vs. control, *p* <0.05; Additional file 2: Figure S1 C, E, F). The morphological changes in the adipose tissue were also similar compared with those in adult mice (Additional file 3: Figure S2). These results indicated that when subjected to starvation or refeeding, the adipose tissue of juvenile mice was influenced more quickly that of adult mice.

## Discussion

Fasting, which is defined as a coordinated set of metabolic changes that spare carbohydrate usage and increase reliance on fat as the energy supply, has been practiced for millennia [9, 44]. In this study, we evaluated the different effects of fasting and subsequent refeeding on various anatomical fat depots involved in thermogenesis, lipid metabolism and lipogenesis, in a long-term fasting and refeeding mouse model.

Several lines of evidence have confirmed [20, 27–30] that food restriction or a very-low-calorie diet could lead to a rapid weight loss and decreased adipose tissue masses in both healthy subjects and obese individuals. In terms of total weight loss, visceral adipose tissue is more sensitive to weight reduction. Mobilization of the subcutaneous depot appears to be less than that of visceral fat during starvation [27–29]. As expected, our results showed that 24 h of fasting mobilized mesenteric WAT preferentially compared with the inguinal WAT, both in terms of fat mass and the morphology of adult mice, which was similar to the results of Ding et al. [23]. However, preferential mobilization of visceral WAT does not mean that the subcutaneous adipose tissues do not respond to food starvation. With the fasting time extended, dramatic changes occurred in the fat mass, as well as the morphology, of subcutaneous depots. When subjected to 72 h of fasting, the consumption of fat masses in the subcutaneous and visceral adipose tissues showed almost similar levels. Our finding might account for the phenomenon that the effect of preferential mobilization of visceral WAT is attenuated with greater weight loss by extended food restriction in human subjects [29]. This observation demonstrated that the mobilization of subcutaneous depots occurred mainly in long-term fasting rather than in short-term fasting.

After refeeding, rapid body weight regain might be attributed to the hyperphagia at the initial phase of refeeding; however, there were no rapid rebounds in body weight compared with the normal level before fasting even after refeeding for 72 h. In addition, no distinct abnormalities in metabolism were observed at this stage. Our data conflicts with previous reports [8, 17] which stated that weight regain after food restriction resulted in accelerated fat storage in adipose tissue, which could result in a rapid repletion and overshoot of body fat. The contradiction might be explained by the different experimental subjects (e.g. human, rats and mice) and the duration of fasting and refeeding. In addition, our results revealed that the speed and extent of recovery of subcutaneous adipose tissues are much greater than those in visceral adipose tissue under refeeding: the visceral WAT were only restored to approximately half the normal levels, while the fat mass of the ingWAT was totally recovered to normal after 72 h of refeeding. These results demonstrated that fasting and refeeding triggered differential responses among adipose tissue depots. Together with the results of Ding et al. [23], we believe that both short-term and long-term fasting and refeeding can lead to a reduction of the ‘metabolically harmful’ visceral adipose tissue.

Although the visceral depot appears to be mobilized preferentially compared with the subcutaneous fat during starvation, the molecular mechanisms by which physiological changes regulate various adipose depots are not fully understood. This study revealed enhanced mitochondrial biogenesis in visceral adipose tissue caused by fasting, as evidenced by the increased mitochondrial numbers and the expression of *Pgc-1α* in visceral adipocytes. *Ucp-1* is not only a BAT-selective gene but also a thermogenic marker [45, 46], which could be detected in some classical “white” adipose tissue depots such as inguinal white adipose tissue [45]. In our present study, *Ucp-1* mRNA levels were markedly reduced in the ingWAT and iBAT after a short-term fasting (24 h), suggesting suppression of thermogenesis in these fat depots, which was partly consistent with a previous study [47]. Interestingly, electron microscopy demonstrated a reduction in mitochondrion numbers in fat depots with stronger thermogenesis (e.g. in the iBAT and ingWAT) and an increase in those with weaker thermogenic adipose tissue (e.g. the eWAT and mWAT). This might, at least in part, contribute to the preferential mobilization of visceral adipose tissue compared with subcutaneous adipose tissue at the initial stage of fasting stage. Accordingly, the differences in mobilization of various fat depots in response to fasting might partly reflect the extent of mitochondrial biogenesis in various adipose tissues.

Studies of genetically manipulated models have suggested that plasticity of the adipose organs was significant in response to changes in environmental cues [48]. A recent study [23] also showed that short-term fasting (24 h) induced a subcutaneous-to-visceral fat switch in mice. However, our morphological results showed that the cell size of inguinal adipocytes was reduced after fasting for 48 h, which suggested that lipid was mobilized in the inguinal adipose tissue at this stage. Therefore, we wondered whether the subcutaneous-to-visceral fat switch would be sustained during extended fasting. The current study confirmed that the expressions of visceral signature genes (*wt1, Tcf21*) were upregulated in the ingWAT after 24 h of fasting. However, these genes showed a dramatic reduction in the ingWAT after fasting for 48 h, which demonstrated strongly that the subcutaneous-to-visceral fat switch might be reversed at this stage. Our results also showed that the expression of genes related to lipolysis (*Atgl, Hsl*) were upregulated sharply with extended fasting time, accompanied by increased fatty acid oxidation, in the subcutaneous depot. Thus, we inferred that once subject to long-term fasting, the subcutaneous-to-visceral fat switch could be reversed to supply energy to compensate for the significant consumption of the visceral adipose tissue. Therefore, the plasticity of the adipose organ in response to starvation might depend on the duration of fasting, which may be evolutionarily important [23, 47]. Our present findings corroborated and extended the results of Ding et al. [23] and collectively suggested that complete fasting consumes the visceral adipose tissue preferentially compared with the subcutaneous depots, which is closely related to the plasticity of various white adipose tissues.

The present results showed a rapid increase in the inguinal adipose tissue mass rather than the visceral depots after refeeding in adult mice. To further investigate lipogenesis after refeeding, markers involved in proliferation and lipogenesis were analyzed. As two main markers for cell increased proliferation, *Ki67* and *Pcna* levels were virtually undetectable in mature mice under fasting or under refeeding conditions, implying the proliferation levels of fat cells in mature mice did not changed although subjected to environmental changes. Actually, Spalding KL et al. [37] had confirmed that the number of fat cells stays constant in adulthood even after marked weight loss, because the number of adipocytes is set during childhood and adolescence. Our results demonstrated that the lipid (eg. triglyceride) stored in fat cell rather than numbers of fat cell had changed when subjected to different feed conditions. Combined with Spalding’s research [37], our results also indicated that mature fat cells didn’t have capacity of proliferation as well as transform to proliferative cells such as multipotential stem cells under various nutritional status. Thus, the recovery of fat is attributed mainly to lipogenesis. As expected, the gene expression of main lipogenic transcription factors (*Pparγ*, *Srebp-1c* and *C/ebpα*) and de novo fatty acid synthesis-related gene (*Fas*) were dramatically upregulated in subcutaneous adipose tissue rather than in visceral depots at the early stage of refeeding. Additionally, the gorging behavior of mice that was triggered by refeeding did not last long, indicating that de novo fatty acid synthesis in adipose tissue dominates over hyperphagia and contributes to the accelerate fat deposition at the initial stage of refeeding. Fasting and refeeding also showed WAT depot-specific changes in the activities of lipogenic enzymes in the fat depots [36]; therefore, the preferential recovery of subcutaneous fat depots might result from the tissue specificity of various adipose tissues. Further study is needed to understand the detailed molecular mechanisms.

The prevalence of obesity is different in the people at different ages [28]. Compared with adolescents, adults are more vulnerable to obesity and related metabolic disorders, while adolescent obesity tends to give priority to the metabolically benign obesity that is generally not associated with cardiovascular risk factors [37–39]. We conducted a similar study in adolescent mice. The data showed age-related alterations in the percentage of body fat in various fat depots: both the fat mass and percentage of body fat of juvenile mice were lower than those in adult mice. There was a remarkable difference in percentage of visceral WAT between juvenile mice and adult mice. Our results also suggested that visceral depots, rather the subcutaneous depots, tend to be affected by age, as is the development of metabolism and internal organs with the growth of body, which is consistent with the study by Wajchenberg [28]. That study showed that visceral adipose tissues grow as humans become older. Accelerated rates of fat recovery resulting in excess adiposity have been reported in adults and children during nutritional recovery after starvation [49]. In the present study, similar phenotypic changes in adipose tissues were observed between juvenile and adult mice when subjected to fasting and refeeding; however, the juvenile mice exhibited a faster deposition of adipose tissue whether under fasting or refeeding condition, which implied that the plasticity of adipose organs in juvenile mice is greater than that in adult mice. A slight dyslipidemia and hyperglycemia was observed in 1-month-old mice after 72 h of ad libitum refeeding, indicating that juvenile mice might be more vulnerable to the influence of the external environment. Notably, the recovery of epididymal adipose tissue was faster in the juvenile mice than in the adult mice under refeeding conditions. One possible reason for this finding could be that the activity and function of fat tissue around the gonads is closely related to the development of the reproductive organs. Taken together, there is an obvious difference in the plasticity of adipose organs at different ages, which might vary according to the anatomy of the adipose tissue. It is tempting to speculate that subcutaneous adipose tissue in juvenile mice might be more sensitive and plastic than that in adult mice.

## Conclusions

In summary, our results demonstrated that fasting induced preferential mobilization of lipids from the mesenteric adipose tissue depot, whereas refeeding induced preferential restoration of adipose tissue from the inguinal depot. These findings confirmed that long-term fasting and refeeding could lead to a reduction of the ‘metabolically harmful’ visceral adipose tissue, as well as highlighting the role of plasticity of adipose organs on different anatomical sites of adipose tissue when subject to environmental changes. A definite trend is evident that modulating the plasticity of adipose organs represents a potential strategy to combat obesity. However, it should be noted that only normal mice were used in the current study and experiments on obese mice are necessary for in future research. Moreover, body fat distribution is controlled by genetic factors [50]; therefore, it is appropriate to explore the mechanism of the effect of fasting and refeeding on various adipose tissues via transcriptome sequencing in future research.

## Additional files

Additional file 1: Table S1. Serum concentrations of metabolites in juvenile mice (1-month-old) subjected to fasting and refeeding. All data are presented as the mean ± SEM. Statistically significant differences: ** p* <0.05; *** p* <0.01; **** p* <0.001; (One-way ANOVA) CON = control juvenile mice, fed ad libitum; F12,F24, F48 and F72 = juvenile mice fasted for 12,24, 48 and 72 h, respectively; R12, R24, R48 and R72 = juvenile mice fasted for 72 h and subsequent fed again for 12, 24, 48 and 72 h, respectively (*n* = 5–12 animals in each group). (DOCX 17 kb)
Additional file 2: Figure S1. Alteration of body weight and fat mass in juvenile mice subjected to fasting and refeeding. 1–month-old mice were fasted for 24, 48 and 72 h (F24, F48 and F72) respectively, and then fed again for 12, 24, 48 and 72 h (R12, R24, R48 and R72) respectively, after 72 h of fast. Body weights (A), food intakes (B) and tissue appearance (C) of inguinal white adipose tissue (ingWAT), epididymal WAT (eWAT), mesenteric WAT (mWAT) and interscapular BAT (iBAT) of mice during different fasting and feeding conditions were detected. Weights of adipose tissues (ingWAT, eWAT, mWAT, iBAT) expressed as a percentage of body weight in juvenile mice (D-G) were also analyzed. All data are presented as mean ± SEM. ** p* <0.05; *** p* <0.01; **** p* <0.001 compared with control mice (Con); ^*a*^ *p <0.05,* ^*b*^ *p <0.001* compared with 72 h-fasting, ^*c*^ *p <0.01* compared with 24 h-refeeding (one-way ANOVA). (DOCX 2179 kb)
Additional file 3: Figure S2. Histomorphological alterations of adipose tissue in juvenile mice subjected to fasting and refeeding. The effects of fasting and refeeding on histomorphological alterations of various adipose tissues in juvenile mice(A-I). The average area of adipocytes (μm^2^) in every 100-mm^2^ area range of various adipose tissues were quantified using Image Pro Plus software (J-M) (*n* = 6–8). Scale bar represents 100 μm, all data are presented as the mean ± SEM. ** p* <0.05; *** p* <0.01; **** p* <0.001 compared with control (one-way ANOVA). (DOCX 2013 kb)

## Abbreviations

ALB: Albumin
*Atgl*: Adipose triglyceride lipase
BK: β-hydroxybutyrate
*C/ebpα*: CCAAT/enhancer binding protein α
*Cpt-1 m*: Carnitine palmitoyltransferase-1 M
*Fas*: Fatty acid synthase
GLU: Glucose
HDL-C: High density lipoprotein cholesterol
*Hsl*: Hormone-sensitive lipase
LDL-C: Low density lipoprotein cholesterol
NEFA: Non-esterified fatty acid
*Pcna*: Proliferating cell nuclear antigen
*Pgc-1α*: Peroxisome proliferator-activated receptor gamma coactivator 1 alpha
*Pparα*: Peroxisome proliferator-activated receptor alpha
*Pparγ*: Peroxisome proliferator activated receptor γ
*Srebp-1c*: Sterol regulatory element binding protein 1c
TC: Total cholesterol
*Tcf2*1: Transcription factor 21
TG: Triglyceride
TP: Total protein
*Ucp-1*: Uncoupling protein 1
*Wt1*: Wilms tumor 1
