# Supplementary material for: Plasticity of adipose tissue in response to fasting and refeeding in male mice
Source: Nutr Metab (Lond). 2017 Jan 5;14:3. doi: 10.1186/s12986-016-0159-x (PMC5217231; doi:10.1186/s12986-016-0159-x)
Supplement: Additional file 1: Table S1. — Serum concentrations of metabolites in juvenile mice (1-month-old) subjected to fasting and refeeding. All data are presented as the mean ± SEM. Statistically significant differences: * p <0.05; ** p <0.01; *** p <0.001; (One-way ANOVA) CON = control juvenile mice, fed ad libitum; F12,F24, F48 and F72 = juvenile mice fasted for 12,24, 48 and 72 h, respectively; R12, R24, R48 and R72 = juvenile mice fasted for 72 h and subsequent fed again for 12, 24, 48 and 72 h, respectively (n = 5–12 animals in each group). (DOCX 17 kb) [file 12986_2016_159_MOESM3_ESM.docx]

**Additional file 2:**

**Figure S2 Histomorphological alterations of adipose tissue in juvenile mice subjected to fasting and refeeding**

**F12**

**Con**

**F24**

**F48**

**F72**

**R12**

**R24**

**R48**

**R72**


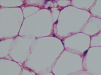

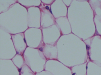

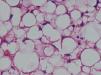

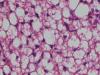

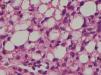

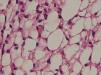

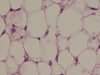

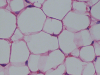

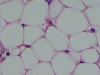

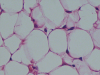

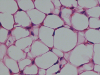

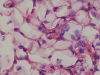

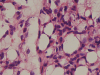

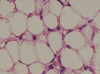

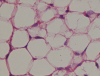

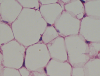

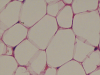

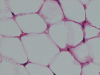

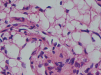

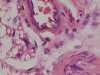

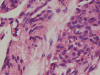

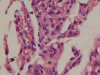

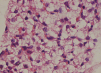

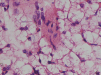

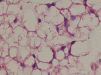

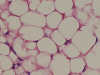

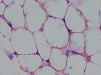

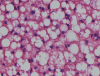

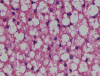

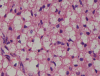

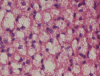

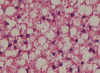

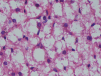

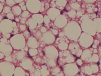

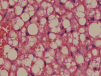

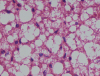


**ingWAT**

**eWAT**

**mWAT**

**iBAT**

**J**

**K**

**L**

**M**

**A**

**B**

**C**

**D**

**I**

**E**

**F**

**G**

**H**

The effects of fasting and refeeding on histomorphological alterations of various adipose tissues in juvenile mice(A-I). The average area of adipocytes (μm^2^) in every 100-mm^2^ area range of various adipose tissues were quantified using Image Pro Plus software (J-M) (n = 6-8). Scale bar represents 100 μm, all data are presented as the mean ± SEM. ** p*<0.05; *** p*<0.01; **** p*<0.001 compared with control (one-way ANOVA).
