# Supplementary material for: Plasticity of adipose tissue in response to fasting and refeeding in male mice
Source: Nutr Metab (Lond). 2017 Jan 5;14:3. doi: 10.1186/s12986-016-0159-x (PMC5217231; doi:10.1186/s12986-016-0159-x)
Supplement: Additional file 2: Figure S1. — Alteration of body weight and fat mass in juvenile mice subjected to fasting and refeeding. 1–month-old mice were fasted for 24, 48 and 72 h (F24, F48 and F72) respectively, and then fed again for 12, 24, 48 and 72 h (R12, R24, R48 and R72) respectively, after 72 h of fast. Body weights (A), food intakes (B) and tissue appearance (C) of inguinal white adipose tissue (ingWAT), epididymal WAT (eWAT), mesenteric WAT (mWAT) and interscapular BAT (iBAT) of mice during different fasting and feeding conditions were detected. Weights of adipose tissues (ingWAT, eWAT, mWAT, iBAT) expressed as a percentage of body weight in juvenile mice (D-G) were also analyzed. All data are presented as mean ± SEM. * p <0.05; ** p <0.01; *** p <0.001 compared with control mice (Con); a p <0.05, b p <0.001 compared with 72 h-fasting, c p <0.01 compared with 24 h-refeeding (one-way ANOVA). (DOCX 2179 kb) [file 12986_2016_159_MOESM1_ESM.docx]

**Additional file 3**

**Table S1. Serum concentrations of metabolites in juvenile mice (1-month-old) subjected to fasting and refeeding.**

|  | **CON** | **F12** | **F24** | **F48** | **F72** | **R12** | **R24** | **R48** | **R72** |
| --- | --- | --- | --- | --- | --- | --- | --- | --- | --- |
| **GLU** | 10.1±1.14 | 7.4±1.16** | 6.24±1.82*** | 5.73±1.99*** | 4.23±1.00*** | 11.71±3.73 | 10.48±2.09 | 9.43±1.21 | 12.61±1.13* |
| **BK** | 0.23±0.1 | 0.82±0.12* | 1.14±0.69** | 1.79±1.02*** | 0.13±0.01 | 0.17±0.07 | 0.15±0.06 | 0.09±0.02 | 0.14±0.03 |
| **TG** | 1.77±0.44 | 0.74±0.25*** | 0.67±0.19*** | 0.7±0.31*** | 0.6±0.07** | 2.55±0.5** | 2.49±0.74** | 1.16±0.19** | 1.26±0.18* |
| **CHOL** | 2.32±0.17 | 2.33±0.37 | 2.4±0.41 | 2.63±0.26 | 2.85±0.36* | 2.1±0.07 | 2.45±0.4 | 2.49±0.07 | 2.31±0.12 |
| **NEFA** | 1.5±0.29 | 2.0±0.17* | 1.63±0.31 | 1.38±0.51 | 1.13±0.07 | 1.11±0.21 | 2.73±0.14*** | 1.45±0.07 | 1.3±0.01 |
| **HDL-C** | 1.75±0.14 | 1.76±0.28 | 1.83±0.32 | 1.97±0.18 | 1.95±0.27 | 1.4±0.05* | 1.56±0.14 | 1.59±0.08 | 1.64±0.09 |
| **LDL-C** | 0.30±0.03 | 0.29±0.04 | 0.34±0.13 | 0.38±0.1 | 0.4±0.06 | 0.17±0.03* | 0.48±0.08** | 0.59±0.04*** | 0.34±0.01 |
| **ALB** | 17.25±1.33 | 18.5±0.7 | 17.63±0.81 | 17.57±0.45 | 16.37±0.81 | 17.33±1.11 | 17.83±1.37 | 17.63±0.49 | 17.27±0.72 |
| **TP** | 48.95±2.69 | 49.23±1.86 | 48.9±3.93 | 47.23±1.61 | 46.8±8.97* | 47.73±3.07 | 48.07±4.68 | 47.37±1.17 | 48.63±2.29 |

All data are presented as the mean ± SEM. Statistically significant differences: ** p*<0.05; *** p*<0.01; **** p*<0.001; (One-way ANOVA) CON = control juvenile mice, fed ad libitum; F12,F24, F48 and F72 = juvenile mice fasted for 12,24, 48 and 72 hours, respectively; R12, R24, R48 and R72 = juvenile mice fasted for 72 hours and subsequent fed again for 12, 24, 48 and 72 hours, respectively (n = 5–12 animals in each group).
