# Supplementary material for: Plasticity of adipose tissue in response to fasting and refeeding in male mice
Source: Nutr Metab (Lond). 2017 Jan 5;14:3. doi: 10.1186/s12986-016-0159-x (PMC5217231; doi:10.1186/s12986-016-0159-x)
Supplement: Additional file 3: Figure S2. — Histomorphological alterations of adipose tissue in juvenile mice subjected to fasting and refeeding. The effects of fasting and refeeding on histomorphological alterations of various adipose tissues in juvenile mice(A-I). The average area of adipocytes (μm2) in every 100-mm2 area range of various adipose tissues were quantified using Image Pro Plus software (J-M) (n = 6–8). Scale bar represents 100 μm, all data are presented as the mean ± SEM. * p <0.05; ** p <0.01; *** p <0.001 compared with control (one-way ANOVA). (DOCX 2013 kb) [file 12986_2016_159_MOESM2_ESM.docx]

**Additional file 1:**

**Figure S1 Alteration of body weight and fat mass in juvenile mice subjected to fasting and refeeding**


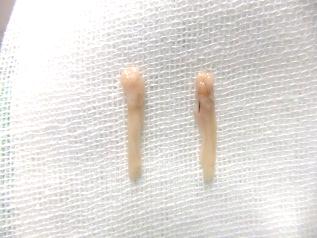

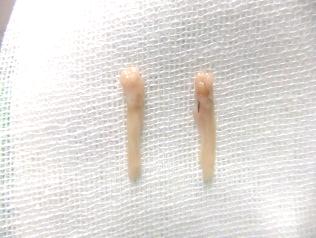

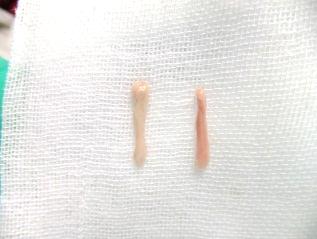

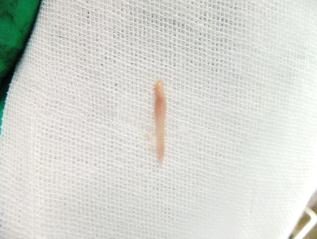

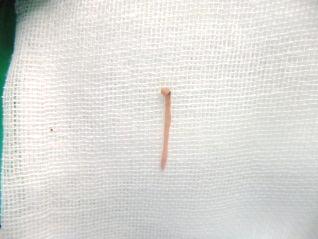

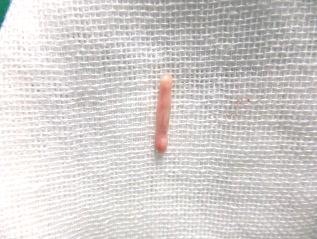

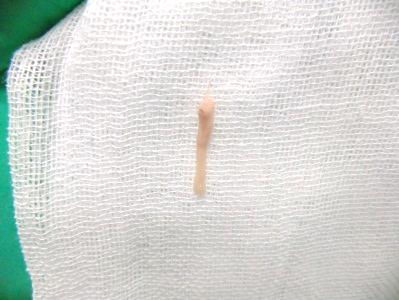

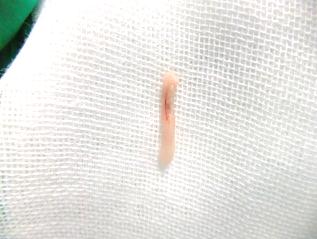

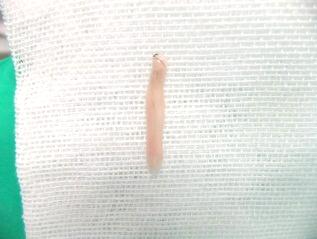

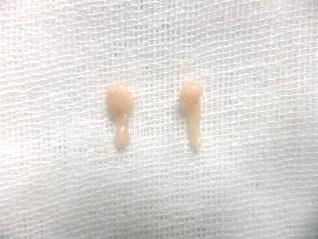

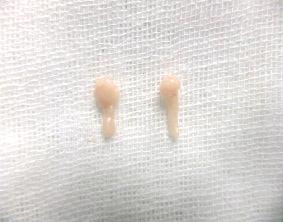

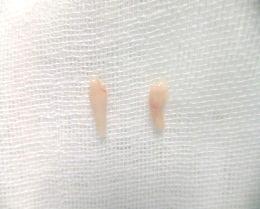

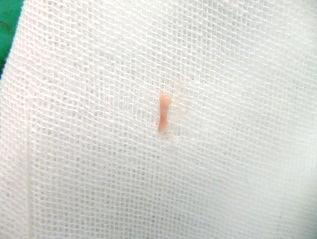

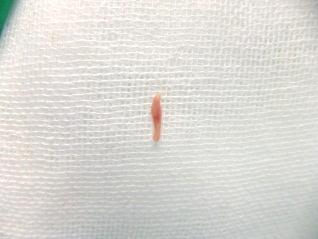

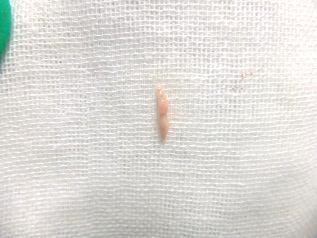

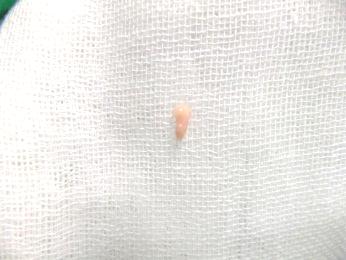

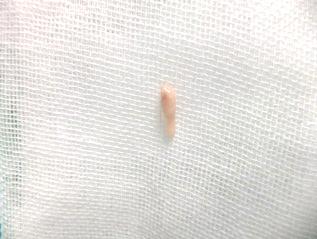

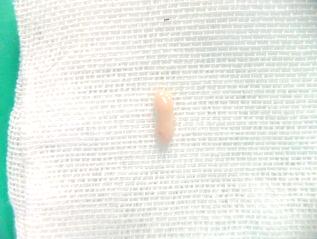

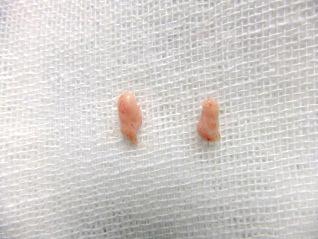

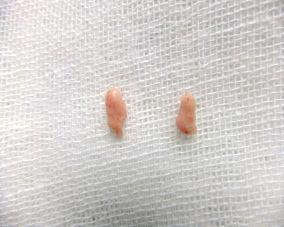

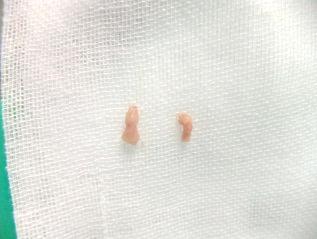

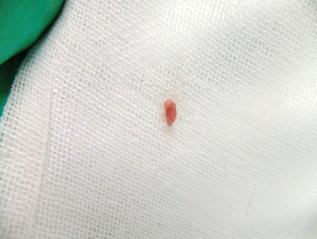

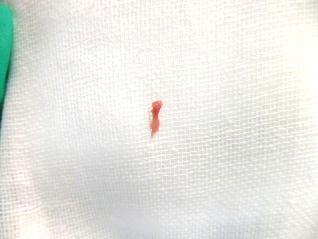

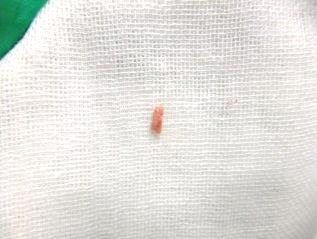

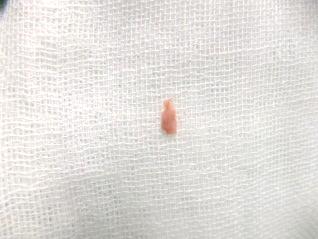

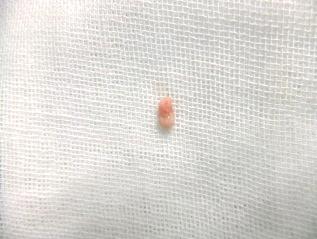

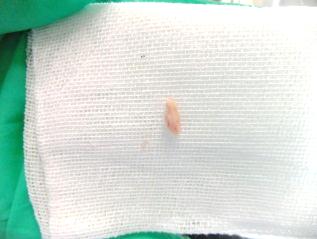

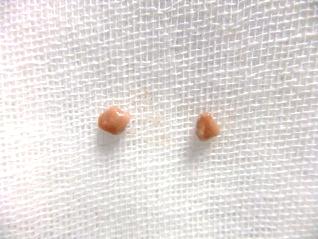

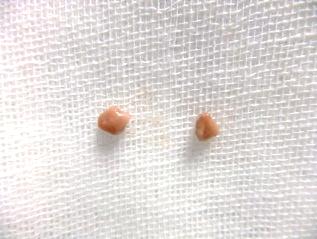

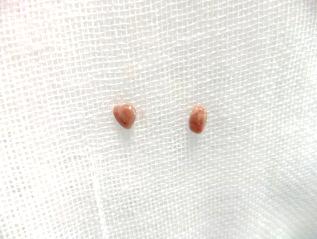

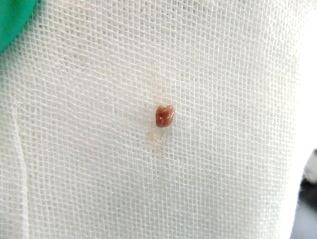

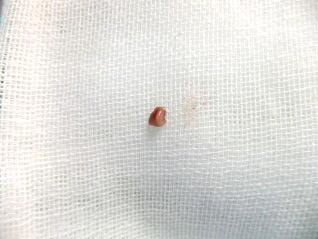

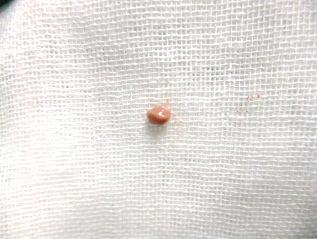

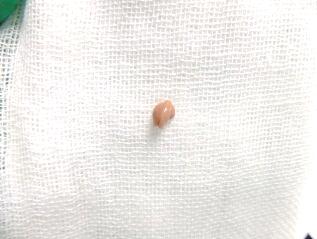

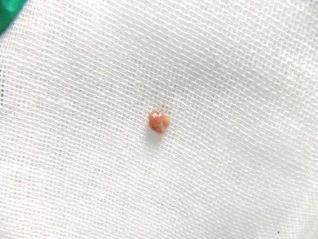

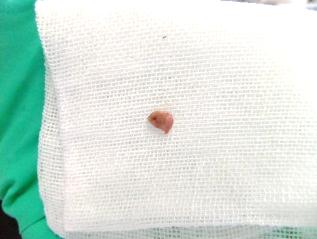


**ingWAT**

**eWAT**

**mWAT**

**iBAT**

**F12**

**F24**

**F48**

**R12**

**R24**

**F72**

**R48**

**R72**

**1-month old**

**CON**

**A**

**B**

**C**

**D**

**F**

**E**

**G**

1–month-old mice were fasted for 24, 48 and 72 hours (F24, F48 and F72) respectively, and then fed again for 12, 24, 48 and 72 hours (R12, R24, R48 and R72) respectively, after 72 hours of fast. Body weights (A) , food intakes (B) and tissue appearance (C) of inguinal white adipose tissue (ingWAT), epididymal WAT (eWAT), mesenteric WAT (mWAT) and interscapular BAT (iBAT) of mice during different fasting and feeding conditions were detected. Weights of adipose tissues (ingWAT, eWAT, mWAT, iBAT) expressed as a percentage of body weight in juvenile mice (D-G) were also analyzed. All data are presented as mean ± SEM. ** p*<0.05; *** p*<0.01; **** p*<0.001 compared with control mice (Con); *^a^ p <0.05, ^b^p <0.001* compared with 72 hours-fasting, *^c^p <0.01* compared with 24 hours-refeeding (one-way ANOVA).
